# De Novo Formation of Left–Right Asymmetry by Posterior Tilt of Nodal Cilia

**DOI:** 10.1371/journal.pbio.0030268

**Published:** 2005-07-26

**Authors:** Shigenori Nonaka, Satoko Yoshiba, Daisuke Watanabe, Shingo Ikeuchi, Tomonobu Goto, Wallace F Marshall, Hiroshi Hamada

**Affiliations:** **1**Graduate School of Frontier Biosciences, Osaka University, Japan,; **2**Department of Biochemistry and Biophysics, University of California, San Francisco, California, United States of America,; **3**CREST, Japan Science and Technology Corporation, Tokyo, Japan,; **4**Molecular Embryology, Department of Biosciences, School of Science, Kitasato University, Kanagawa, Japan,; **5**Department of Mechanical Engineering, Tottori University, Tottori, Japan; Wellcome Trust Sanger InstituteUnited Kingdom

## Abstract

In the developing mouse embryo, leftward fluid flow on the ventral side of the node determines left–right (L-R) asymmetry. However, the mechanism by which the rotational movement of node cilia can generate a unidirectional flow remains hypothetical. Here we have addressed this question by motion and morphological analyses of the node cilia and by fluid dynamic model experiments. We found that the cilia stand, not perpendicular to the node surface, but tilted posteriorly. We further confirmed that such posterior tilt can produce leftward flow in model experiments. These results strongly suggest that L-R asymmetry is not the descendant of pre-existing L-R asymmetry within each cell but is generated de novo by combining three sources of spatial information: antero-posterior and dorso-ventral axes, and the chirality of ciliary movement.

## Abstract 2

Left-right asymmetry in the developing mouse embryo is established by leftward fluid flow. Here the authors demonstrate how a posterior tilt of beating cilia creates this unidirectional fluid flow.

## Introduction

The first known left–right (L-R) symmetry-breaking event in mouse development occurs at the ventral surface of the node at embryonic day 8.0 (E8.0) [1]. Previous studies showed that hundreds of monocilia on the node rotate in a clockwise direction and generate leftward flow of extraembryonic fluid, referred to as nodal flow [2]. The direction of this flow determines which side will be the left, because the L-R decision is reversed when the flow direction is reversed by imposing artificial flow [3]. Although nodal flow is clearly important, at least in the mouse, several questions remain to be answered. First, it is unknown how the nodal flow works to control the L-R decision. Two models have been proposed. The nodal flow may transport a signaling molecule toward the left side [2]. Alternatively, mechanical stress generated by the flow may be sensed by a mechanosensor on node cells [4,5]. Currently, there is no direct evidence for one model or the other. Second, it is not easy to envisage how unidirectional flow is generated by rotating cilia, because a rotational movement should cause a vortex, and the sum of the vortices should result in a circular flow around the edge of the node. In practice, however, we never observe such a vortical flow but only a linear leftward flow. Although theoretical models have been proposed [6] in which rotational movement can result in unidirectional flow, their validity has not been tested experimentally. Finally, it remains to be clarified whether the flow plays a conserved role among vertebrates, although fluid flow has been detected in the Kupffer's vesicle of zebrafish embryo, which may be equivalent to the mode node [7].

A recent paper by Cartright et al. [6], describing theoretical fluid dynamics simulations of nodal cilia rotation, showed that a linear flow could result if the rotation axis of the cilia has a posterior tilt. Their simulation predicted a three-layered flow pattern: Rotational movement of cilia directly makes a diphasic flow that is leftward at the level of the cilia tips and rightward closer to the node floor. The third layer is a boundary-induced return flow to the right, predicted on the basis of conservation of mass if Reichert's membrane seals the cavity surrounding the node. This theoretical simulation can explain how rotational motion is converted to linear flow.

An interesting feature of the Cartwright model is the fact that the leftward flow that is usually observed in the node is balanced by two layers of rightward flow. Boundary-induced rightward flow across the top of the node is necessary because in utero development occurs within a closed volume inside of Reichert's membrane. Such a rightward flow, however, appears to be unnecessary for generating L-R asymmetry because wild-type embryos with Reichert's membrane removed develop normal situs, and an immotile cilia mutant (inversus viscerum [iv]) [8] can establish L-R asymmetry in artificial flow experiments [3] in which the flow pattern is completely linear and unidirectional. More problematic is the prediction that a second layer of active rightward-directed flow should occur near the node floor. This rightward flow near the surface of the node has never been observed in the mouse embryo [2,3,9].

**Figure 1:**
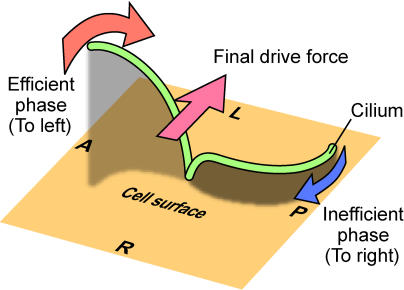
Flow Generation Mechanism Circular clockwise motion of a cilium can generate directional leftward flow if its axis is not perpendicular to the cell surface but tilted posteriorly. Due to distance from the cell surface, a cilium in the leftward phase (red arrow) drags surrounding water more efficiently than the rightward phase (blue arrow), resulting in leftward force in total (purple arrow). See text for details. The rotating cilium is drawn slightly bent due to viscous resistance, as seen in Video S1.

Brokaw [10] has pointed out that surface interactions could produce a leftward flow from rotational ciliary movement without generating any significant rightward flow near the surface. Usually fluid contacting a solid wall will not move (the so-called no-slip boundary condition), and this static fluid layer should impede overlying fluid from being dragged by the cilia due to viscous force [11]. This effect should be greatest near the surface. Cartwright et al. [6] mention that the flow close to the floor is slower due to this effect, but in fact if this effect is great enough, it could effectively produce unidirectional propulsion toward the left (Figure 1). As proposed by Brokaw [10], if a clockwise rotating cilium has posterior tilt, it passes closest to the surface during the rightward phase and farthest from the surface during the leftward, and thus should drag surrounding water the least efficiently during rightward motion and the most efficiently during leftward motion. In total, while at a very small scale, the local flow will have a rotational component; at large scales the leftward flow will predominate over the rightward, and the rightward flow cannot make a continuous flow layer. Brokaw's model can account for the fact that cilia-induced rightward flow was never observed in our previous bead-tracking experiments [2,9], thus reconciling the essential aspects of Cartwright's tilted cilia model with the experimental data that rightward flow near the surface is not observed in vivo. Exploitation of surface effects, by dragging motile limbs close to the surface during a return stroke, is a common strategy for generating directed motile forces in small organisms such as copepods [12]. Brokaw's model proposes a fundamentally similar strategy for generating nodal flow. Indeed, this model is based on previous discussions of propulsion by ciliary movement in which return strokes occur near a surface [13], and thus the model does not invoke any unusual fluid dynamics beyond what has already been described in other systems.

In this paper, we have studied how unidirectional flow is generated by the rotational movement of node cilia, by testing the validity of Brokaw's model [10]. Our data suggest that node cilia do not protrude perpendicular to the node surface but are tilted posteriorly, and that this posterior tilt is responsible for generating the leftward flow. Therefore, L-R symmetry breaking is achieved by integration of the pre-existing positional information, anterio-posterior and dorsoventral information.

## Results/Discussion

### Node Cilia Are Tilted Posteriorly

**Figure 2:**
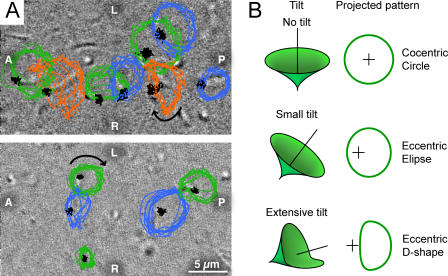
Trajectory of Node Cilia Movement (A) Trace of node cilia in enhanced DIC images after background subtraction. Positions of root are indicated in black, and tip in blue, green, and orange. Most cilia have a pattern consistent with the projection of a tilted cone (blue and green, see text) whereas some cilia move in a D-shape (orange). A, P, L, and R refer to anterior, posterior, left, and right sides of the node, respectively. The direction of cilia rotation was clockwise (arrows). (B) Relationship between essentially rotatory movement of cilia and their projected images at various tilt angles.

To test the validity of Brokaw's theoretical model experimentally, we first examined whether the node cilia are really tilted posteriorly. Trajectories of the cilia were tracked by a high-speed camera attached to a differential-interference contrast (DIC) microscope, followed by image processing to enhance subtle contrast (Figure 2A, Video S1). If a cilium is rotating around an axis that is directed straight up (that is, toward the ventral axis), the movements of the tip would describe a circle with the root of the cilium located in the center (Figure 2B, top). In contrast, if a cilium is tiltedposteriorly, the tip should appear as an ellipse and the position of the root should be displaced from the center anteriorly (Figure 2B, middle). The majority of observed cilia had exactly such a pattern. Some cilia traced out the shape of a “D”, suggesting that the trajectory of the tip was not strictly circular. This shape resembled that of *Paramecium* cilia [14,15]. *Paramecium* cilia have an effective stroke and a recovery stroke that take place in different planes, resulting in a D-shaped pattern when viewed from the top. The change in bending plane between the effective and recovery strokes is thought to be mediated by rotation of the central pair microtubules in *Paramecium* cilia [16]. However, the nodal cilia are the 9+0 type, which lacks the central pair [17], making it unlikely that the node cilia could move with a *Paramecium*-like two-phase beat pattern. Furthermore, *Paramecium* cilia usually beat in a D-shape pattern [14], not in the elliptical paths that were observed for the majority of node cilia.

An alternative interpretation of the observed D-shape pattern is to consider the D-shape pattern as simply a rotating cilium whose axis is tilted at a sufficiently acute angle relative to the surface that the tip cannot trace a circle without hitting the surface (Figure 2B, bottom). In such a case, the curved portion of the “D” results when the cilium scrapes along the surface of the node, whereas the straight portion results when the cilium rotates up away from the surface. Given the orientation of the “D” shapes observed, it is notable that in all cases it appears that the movement away from the surface occurs during the leftward phase, whereas the scraping movement along the surface is during the rightward phase. As discussed by Brokaw [10], the no-slip boundary condition would then predict that during this rightward phase, little fluid would be moved by the cilium due to its proximity with the immobilized fluid layer at the surface. Therefore, the D-shaped trajectories of moving cilia emphasize the potential for surface interactions to have a major effect on flow generation, arguing strongly that such interactions should never be neglected.

**Figure 3:**
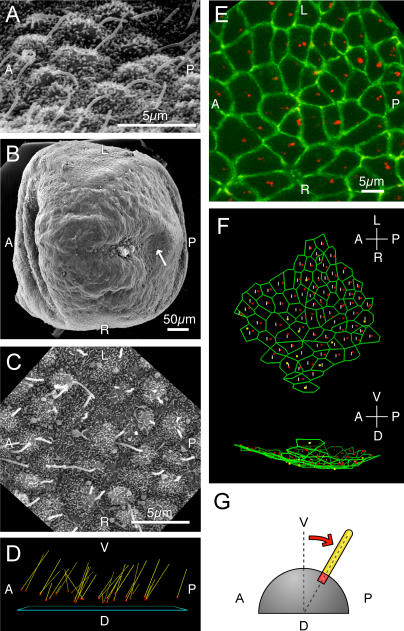
Node Cilia Are Posteriorly Tilted and Positioned (A) Scanning electron micrograph of the wild-type node. Note that cilia emanate from the posterior part of the cells. The view angle is about 30° with respect to the horizontal line. (B, C) Scanning electron micrograph of the iv/iv node. (C) is a high-magnification picture of the region in (B) indicated by an arrow. (D) Deduced tilt of iv/iv node cilia after stereography from multiple-tilt scanning electron micrograph images. Yellow lines indicate cilia, red dots their root positions, and a blue square a plane best-fit to the node surface. When we calculated the tilt of the cilia, we separated the tilt into A-P (anterior–posterior) and L-R components. The average tilt was 26.6° in A-P axis (toward the posterior) and 0.06° in L-R axis (towards the right). (E) Immunofluorecence image of node cells shown as projection of 3D confocal data stack. Basal bodies and cell boundaries are shown by immunofluorescence against γ-tubulin (red) and ZO-1 (green), respectively. (F) 3D reconstruction of (E) viewed from ventral side (top) and right side (bottom), showing posterior bias of basal body positions. White lines divide the cells into the anterior and the posterior halves. Basal bodies located in the anterior and the posterior are shown in yellow and red, respectively. (G) Speculative interpretation of posterior bias of basal bodies in orientation and position of the node cilia. Because the node cells are somewhat rounded, if basal bodies were located at the posterior part of these cells, it would result in posteriorly tilted cilia even though the basal bodies remain perpendicular to the plasma membrane.

Second, we measured tilt of the cilia from scanning electron microscopy images (Figure 3). Posterior tilt is immediately apparent from side-viewed images (Figure 3A); however we performed three-dimensional (3D) stereographic analysis to eliminate any possibility that the tilt might be an illusion caused by the viewpoint. We used iv/iv mutants for stereographic analysis because, in the absence of dynein-driven motility [8], we expect that the direction of the rigid cilia [9] would indicate the orientation of the basal body and therefore represent the axis of the conic pathway around which wild-type cilia would rotate. We measured 225 cilia from six different embryos, and by comparing the orientation of each cilium with the orientation of a plane best-fit to the node surface, we found that the cilia showed an average 26.6° tilt towards the posterior. The individual average tilt angles within each embryo ranged from 15° to 35° but were always directed towards the posterior (Figure 3D), as required by theoretical models for flow generation by tilted rotation [6,10]. The fact that the same orientation is observed in multiple embryos fixed independently argues that the tilt is not due to artifacts generated during sample preparation. In the scanning electron microscopy images, we also noted that most of the node cilia protrude from the posterior region of the node pit cells. This was the case, not only for iv/iv cilia (Figure 3C), but also for wild-type cilia (Figure 3A), and was confirmed by immunofluorescent analysis of basal bodies (Figure 3E and 3F). Out of 83 basal bodies examined, 73 were located in the posterior half of the cells (Figure 3F). This posteriorly oriented location of the cilia within cells whose surface is rounded may be responsible for the posterior tilt of the cilia (Figure 3G). Additionally, another paper recently published also reports posterior tilt of the node cilia [18].

### Tilted Rotation Can Generate Unidirectional Flow

**Figure 4:**
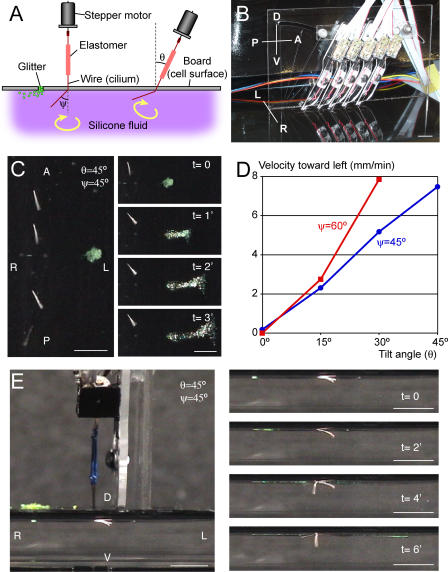
Experimental Fluid Dynamics Model of the Posterior Tilt Mechanism (A) Schematic representation of the model. Wires driven by stepper motors stir silicone fluid of 30,000 centipoise. Note that the fluid surface is in contact with the acrylic board. Resulting flow is visualized as movement of glitter particles inserted through holes on the board. Wires are connected to motor axles by elastomer connectors in order to smoothen the stepwise motion of the motors. The path of the wires is completely specified by the two angles θ and ψ, as shown in the diagram. (B) Photograph of the model. Corresponding directions are indicated. (C) Leftward flow. Flow generated by five rotating wires (visible as white lines in photo) drives glitter towards the left at the depth of 3 mm from the surface. Trajectory of the glitter becomes curved far from the wires (t = 3′) because the glitter has reached the chamber's wall. Scale = 1 cm. (D) Flow velocity (leftward component) as a function of tilt and bend angles. The flow velocity shown here represents the flow speed at the depth of 3 mm. The flow is most efficient when the path of the wire is tangential to the board surface during part of its rotation (i.e., ψ + θ = 90°). (E) Leftward flow near the surface. Glitter was injected on the right side at the surface of the fluid (no more than 1-mm depth). Only leftward flow was observed. Scale = 1 cm.

Finally we confirmed experimentally that tilted rotation can generate leftward flow without any detectable rightward flow near the surface, as predicted by considerations of surface effects on flow [10,13]. For this purpose we constructed a mechanical model of 1000 × in scale: Five wires (0.2-mm diameter, 6-mm long) rotate with conical pathways and stir viscous silicone fluid of 30,000 centipoise (Figure 4A and 4B). As shown in Figure 4C (Video S2) and 4D, this model can indeed generate leftward flow from ciliary rotation. The velocity of the leftward flow depends on the tilt angle: A faster flow was generated when the tilt angle was larger (Figure 4D). Closer to the surface, the flow was still leftward (Figure 4E; Video S3), indicating that any rightward flow that may occur is insignificant in magnitude relative to the leftward flow, consistent with our predictions based on the no-slip boundary condition. Unidirectional flow was not formed if the rotation axis of the wires was not tilted (Video S4).

To simulate fluid dynamics at different size scales, it is important to consider the Reynolds number *(Re).* *Re* is the ratio between inertial force to viscous force and is very low in microscopic phenomena, i.e., viscous force is dominant and inertial force negligible. The *Re* for ciliary rotation *(Re_c_)* has been calculated as *Re_c_* ≍ 5 × 10^−4^ in node cilia [6]. From Figure 2A we estimate the length of cilia is about 6 μm, then *Re_c_* ≍ 1 × 10^−3^. In our mechanical model, *Re_c_* = 2 × 10^−2^. Although the two Reynolds numbers are different, they both fall in the “creeping flow” regime of low *Re* in which inertial forces are negligible, thus the same laws of motion will govern fluid behaviour in both cases. To confirm this, another model was constructed that had a single cilia with *Re_c_* = 4.6 × 10^−4^. This model also produced leftward flow in the layer close to the surface (Video S5).

### Concluding Remarks

Here we demonstrated that the node cilia are tilted posteriorly and that rotation of posteriorly tilted cilia can produce leftward flow instead of vortices. Therefore, L-R asymmetry of the body without preceding L-R asymmetry can be explained by relying on information obtained from the antero-posterior and dorso-ventral axes and the chirality of ciliary movement. In this model, dorso-ventral axis information is used to orient the basal bodies along the apical–basal axis of ventral node cells, and antero-posterior axis information is used to tilt this axis towards the posterior. Finally, the inherent chirality of the cilium is used to bias the rotation direction clockwise, thus leading to directed flow.

Brown and Wolpert [19] hypothesized an “F-molecule” for establishing L-R asymmetry in vertebrate development. A chiral structure would create the first difference along L-R axis by orienting itself with the other axes, then this nanometer scale asymmetry would be amplified to cellular and organism scale by directional transport of another molecule. We propose that the node cilia fulfill the criteria for the F-molecule in the mammalian case.

## Materials and Methods

### 

#### Motion analysis of live node cilia

E8.0 mouse embryos were dissected as previously described [2]. Motion of the cilia was recorded with a high-speed camera at 200 frames/s (HAS-200EX, Ditect, Tokyo, Japan) attached to a DIC microscope (Axiophot-2, Zeiss, Oberkochen, Germany) with 63× water-immersed objective and 2.5× intermediate lens. The background of the images was subtracted and the contrast enhanced using a custom-made Ruby script. All the traceable cilia were digitized at roots and tips using free Object-Image software developed by Norbert Vischer (http://simon.bio.uva.nl/object-image.html)

#### Scanning electron microscopy and stereography

E8.0 mouse embryos were processed using standard procedures [2] and observed by a scanning electron microscope (S-800, Hitachi, Tokyo, Japan). Sets of photographs were taken with 0° and plus or minus 10° tilts at 6,000× magnification for cilia and one at 200× for the whole embryo to measure the direction of the antero-posterior axis. One embryo was excluded from further analysis because it had severely curved cilia, presumably due to a fixation artefact. Photographs were digitized and used to calculate 3D coordinates using custom software written within Matlab (MathWorks, Natick, Massachusetts, United States.). Because the high-magnification photographs cover only a small part of the node, the surface of the node can be approximated as a plane within each dataset. Tilt angles were calculated as the angle between each cilium and the normal vector to the planar surface. Results were visualized with free Rotater software written by Craig Kloeden (http://casr.adelaide.edu.au/rotater/).

#### Immunofluorescence analysis

As in our previous paper [20], node tissue fixed with ice-cold acetone was stained by a rabbit polyclonal antibody to γ-tubulin (Sigma, St. Louis, Missouri, United States) and a mouse monoclonal antibody to ZO-1 kindly provided by S Tsukita [21]. Confocal images were obtained with a LSM 510 confocal microscope (Zeiss) and analyzed using Object-Image software. Lines dividing the cells into the anterior and the posterior halves were determined as the line that passes through the center of mass of the cell borders and is perpendicular to antero-posterior axis of the embryo.

#### Fluid dynamic experiments using a mechanical model

Copper wire of 0.2 mm diameter was bent at 45° or 60°, then cut so that the length of wire protruding into the silicone fluid would be 6 mm. In the five-wire model, the wires were connected to small stepper motors (CAM-60, Canon, Tokyo, Japan) and rotated at 2.7 Hz in silicone fluid of 30,000 centipoise (Clearco, Bensalem, Pennsylvania, United States) in a rectangular Petri dish (AW2000, Eiken-Kizai, Tokyo, Japan) of inner dimensions 138 mm × 98 mm × 13 mm. To visualize fluid movement, glitter was directly applied to the surface up to a depth of 1 mm or 3 mm. In the single-wire model, a wire bent at 45° was connected to a DC motor with a reduction gearbox and rotated at 5.6 × 10^−2^ Hz. Glitter was applied up to a depth of 1 mm.

## Supporting Information

Video S1 Motion of Node Cilia Described in Figure 2A The video is 20 times slower than real time. See Figure 2A for details.
(4.8 MB MOV).

Video S2 Flow Generated by the Mechanical Model Described in Figure 4C The video speed is 30 times real time.
(64 KB MOV).

Video S3 Leftward Flow Generated near the Surface Shown in Figure 4E The video speed is 30 times real time.
(370 KB MOV).

Video S4 Flow Resulting in a Big Vortex around Rotating Wires, Rather than Linear Flow, When Their Rotation Axis Is Not Tilted (θ = 0°, ψ = 45°) The video speed is 30 times real time.
(398 KB MOV).

Video S5 Leftward Flow Generated by a Single-Wire Model with a Lower *Re_c_*
The *Re_c_* = 4.6 × 10^−4^. The video speed is 480 times real time.
(266 KB MOV).

## Abbreviations

DIC: differential-interference contrast
iv: inversus viscerum
L-R: left–right
Re: Reynolds number
3D: three-dimensional

## References

[pbio-0030268-b01] Hamada H, Meno C, Watanabe D, Saijoh Y (2002). Establishment of vertebrate left-right asymmetry. Nat Rev Genet.

[pbio-0030268-b02] Nonaka S, Tanaka Y, Okada Y, Takeda S, Harada A (1998). Randomization of left-right asymmetry due to loss of nodal cilia generating leftward flow of extraembryonic fluid in mice lacking KIF3B motor protein. Cell.

[pbio-0030268-b03] Nonaka S, Shiratori H, Saijoh Y, Hamada H (2002). Determination of left-right patterning of the mouse embryo by artificial nodal flow. Nature.

[pbio-0030268-b04] Tabin CJ, Vogan KJ (2003). A two-cilia model for vertebrate left-right axis specification. Genes Dev.

[pbio-0030268-b05] McGrath J, Somlo S, Makova S, Tian X, Brueckner M (2003). Two populations of node monocilia initiate left-right asymmetry in the mouse. Cell.

[pbio-0030268-b06] Cartwright JH, Piro O, Tuval I (2004). Fluid-dynamical basis of the embryonic development of left-right asymmetry in vertebrates. Proc Natl Acad Sci U S A.

[pbio-0030268-b07] Essner JJ, Amack JD, Nyholm MK, Harris EB, Yost HJ (2005). Kupffer's vesicle is a ciliated organ of asymmetry in the zebrafish embryo that initiates left-right development of the brain, heart and gut. Development.

[pbio-0030268-b08] Supp DM, Witte DP, Potter SS, Brueckner M (1997). Mutation of an axonemal dynein affects left-right asymmetry in inversus viscerum mice. Nature.

[pbio-0030268-b09] Okada Y, Nonaka S, Tanaka Y, Saijoh Y, Hamada H (1999). Abnormal nodal flow precedes situs inversus in iv and inv mice. Mol Cell.

[pbio-0030268-b10] Brokaw CJ (2005). Computer simulation of flagellar movement IX. Oscillation and symmetry breaking in a model for short flagella and nodal cilia. Cell Motil Cytoskeleton.

[pbio-0030268-b11] Liron N (1996). Stokes flow due to infinite arrays of stokeslets in three dimensions. J Eng Math.

[pbio-0030268-b12] Childress S, Koehl MAR, Miksis MJ (1987). Scanning currents in Stokes flow and the efficient feeding of small organisms. J Fluid Mech.

[pbio-0030268-b13] Blake JR, Sleigh MA (1974). Mechanics of ciliary locomotion. Biol Rev Camb Philos Soc.

[pbio-0030268-b14] Tamm SL (1972). Cillary motion in *Paramecium* A scanning electron microscope study. J Cell Biol.

[pbio-0030268-b15] Blake J (1974). Hydrodynamic calculations on the movements of cilia and flagella. I. *Paramecium*. J Theor Biol.

[pbio-0030268-b16] Omoto CK, Gibbons IR, Kamiya R, Shingyoji C, Takahashi K (1999). Rotation of the central pair microtubules in eukaryotic flagella. Mol Biol Cell.

[pbio-0030268-b17] Bellomo D, Lander A, Harragan I, Brown NA (1996). Cell proliferation in mammalian gastrulation: The ventral node and notochord are relatively quiescent. Dev Dyn.

[pbio-0030268-b18] Okada Y, Takeda S, Tanaka Y, Belmonte JC, Hirokawa N (2005). Mechanism of nodal flow: A conserved symmetry breaking event in left-right axis determination. Cell.

[pbio-0030268-b19] Brown NA, Wolpert L (1990). The development of handedness in left/right asymmetry. Development.

[pbio-0030268-b20] Watanabe D, Saijoh Y, Nonaka S, Sasaki G, Ikawa Y (2003). The left-right determinant Inversin is a component of node monocilia and other 9+0 cilia. Development.

[pbio-0030268-b21] Itoh M, Nagafuchi A, Yonemura S, Kitani-Yasuda T, Tsukita S (1993). The 220-kD protein colocalizing with cadherins in non-epithelial cells is identical to ZO-1, a tight junction-associated protein in epithelial cells: cDNA cloning and immunoelectron microscopy. J Cell Biol.

